# Perforation of a Pyometra: A Case Report

**DOI:** 10.7759/cureus.77719

**Published:** 2025-01-20

**Authors:** Rutu Vadhadiya, Kanchan S Dwidmuthe, Anuja Bhalerao

**Affiliations:** 1 Department of Obstetrics and Gynaecology, NKP Salve Institute of Medical Sciences and Research Centre, Nagpur, IND

**Keywords:** acute abdomen, exploratory laparotomy, pneumoperitoneum, pyometra, total abdominal hysterectomy, uterine perforation

## Abstract

Pyometra, characterized by the accumulation of purulent material in the uterine cavity, can result in spontaneous uterine perforation, which is a rare but life-threatening complication. This report presents a unique case of uterine perforation as a result of pyometra in a postmenopausal woman. A 52-year-old postmenopausal woman presented to the emergency department with severe abdominal pain, fever, nausea, and vomiting. Clinical suspicion was initially directed toward acute appendicitis. However, imaging revealed pneumoperitoneum with multiple air pockets, suggesting intestinal perforation. Exploratory laparotomy uncovered no bowel perforation but revealed a 2x2 cm uterine fundal perforation with pus drainage. A total abdominal hysterectomy with bilateral salpingo-oophorectomy was performed. Histopathology confirmed acute necrotizing inflammation extending into the myometrium, with no malignancy detected. Therefore, early diagnosis and surgical intervention, including hysterectomy, are critical for reducing the high morbidity and mortality associated with this condition. Hence, this case underscores the importance of considering uterine perforation as a differential diagnosis in elderly postmenopausal women presenting with acute abdomen for which prompt surgical management is essential to ensure a favorable outcome.

## Introduction

Pyometra is the collection of purulent material in the uterus that results from inadequate uterine cavity drainage [1]. Diffuse peritonitis, a rare consequence with a reported frequency of 0.01-0.05%, can result from spontaneous uterine perforation caused by pyometra with associated uterine wall necrosis. In postmenopausal women, its frequency might reach 13.6% [1,2]. Genital tract malignancy is the primary cause of pyometra, while other contributing factors include retained intrauterine device (IUD) or foreign body, radiation therapy, endometrial lesions, senile cervicitis puerperal infection, fibroid, and congenital cervical anomaly [3].

The uterus has a significant circulatory supply, which makes uterine perforations rare. Gradually, pyometra develops and causes the uterus to expand. It also causes degenerative changes that can occasionally result in the uterine wall sloughing and its contents spilling into the abdominal cavity [4]. Majorly, uterine perforations occur at the fundus; however, they may present posteriorly or anteriorly [5]. The pneumoperitoneum is caused during laparotomy and laparoscopy, hollow viscus perforation, and penetrating trauma. Pneumoperitoneum, however, is an extremely uncommon presentation in uterine perforations [6]. This report presents a case of uterine perforation in a 52-year-old postmenopausal woman who presented to the emergency department with severe abdominal pain, fever, nausea, and vomiting.

## Case presentation

Patient information

A 52-year-old woman presented to the obstetrics and gynecology emergency department with the chief complaints of fever and periumbilical pain that had been persistent since four days, along with vomiting and nausea. The patient reported that she had constipation for three days. Additionally, the patient had a history of bronchial asthma. Moreover, the woman was post-menopausal since seven years. The patient reported that she had six pregnancies, resulting in four living children and two neonatal/infant deaths (G6P6L4D2), and tubal ligation was also performed 22 years ago.

Clinical examination

The patient's vital signs were stable. On physical examination, the abdomen was flat, the umbilicus was centrally placed, and no dilated veins or discoloration was observed. There was tenderness on the right iliac fossa (RIF) and left iliac fossa (LIF) with guarding and absent intestinal sounds, and no rigidity and organomegaly were recorded. On percussion, a tympanic note was present over the abdomen. On per-speculum examination, the cervix and vagina were flushed with minimum vaginitis along with a small polyp from the endocervix. On per-vaginal examination, the uterus was depicting an impression of eight weeks' size. Additionally, the cervix was short and hard in consistency. Based on the clinical impression of acute appendicitis, the patient was treated with an injection pan, paracetamol, and ceftriaxone, and was directed for further investigations.

Diagnostic assessment

**Figure 1:**
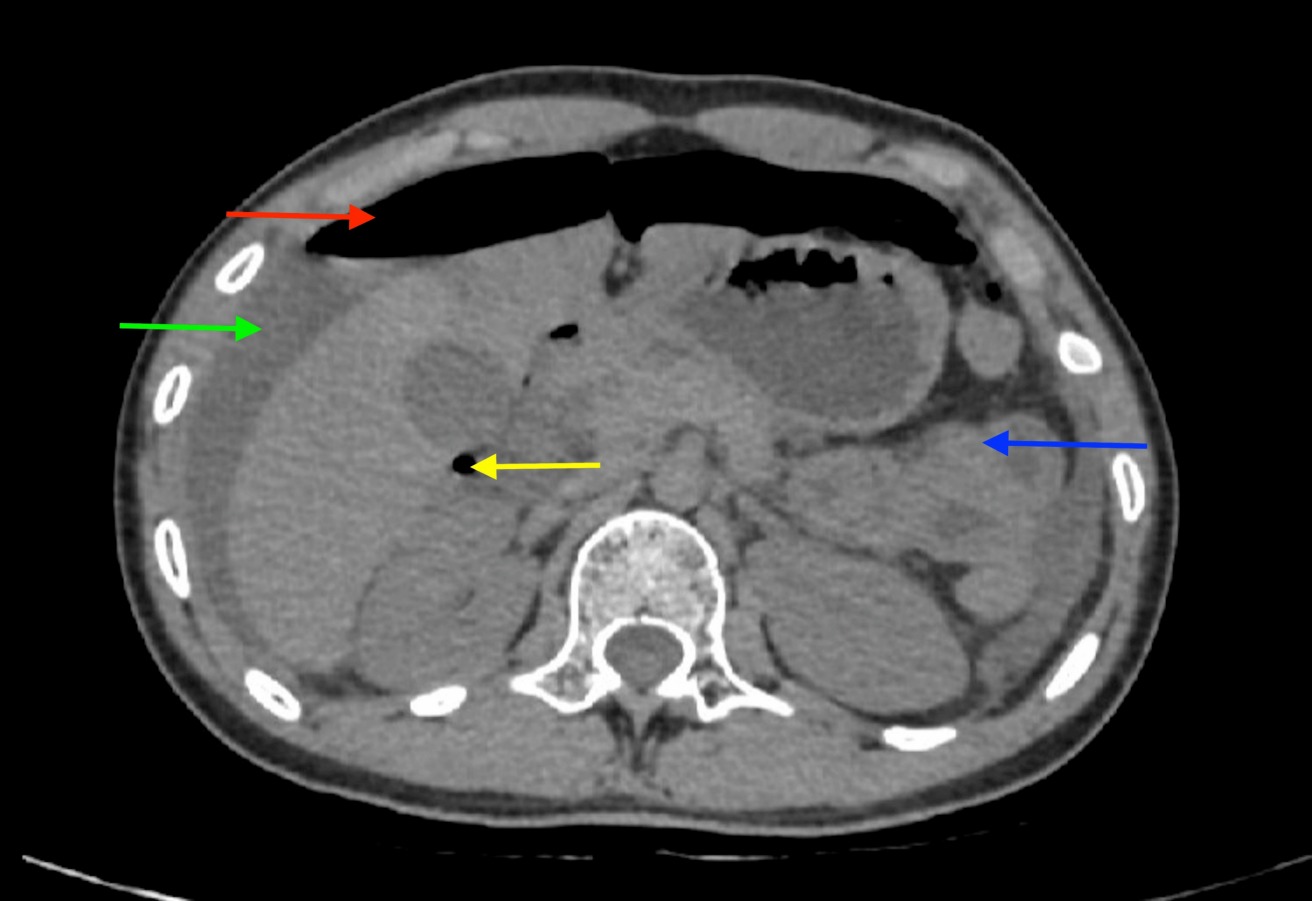
Computed tomography of the abdomen The red arrow shows pneumoperitoneum, yellow arrow shows air pockets, blue arrow shows collapsed ileal loops, and green arrow shows moderate free fluid.

For diagnostic confirmation, a 2D echo and chest radiograph were performed, which were found to be normal. The ultrasonography (USG) report of the abdomen revealed distended loops in LIF and the left lumbar region, with fecal matter and raised vascularity along with moderate ascites. Computed tomography (CT) of the abdomen confirmed pneumoperitoneum with multiple air pockets, collapsed ileal loops, and moderate free fluid in the abdomen and pelvis (Figure 1), strongly indicating an intestinal perforation.

Therapeutic intervention

**Figure 2:**
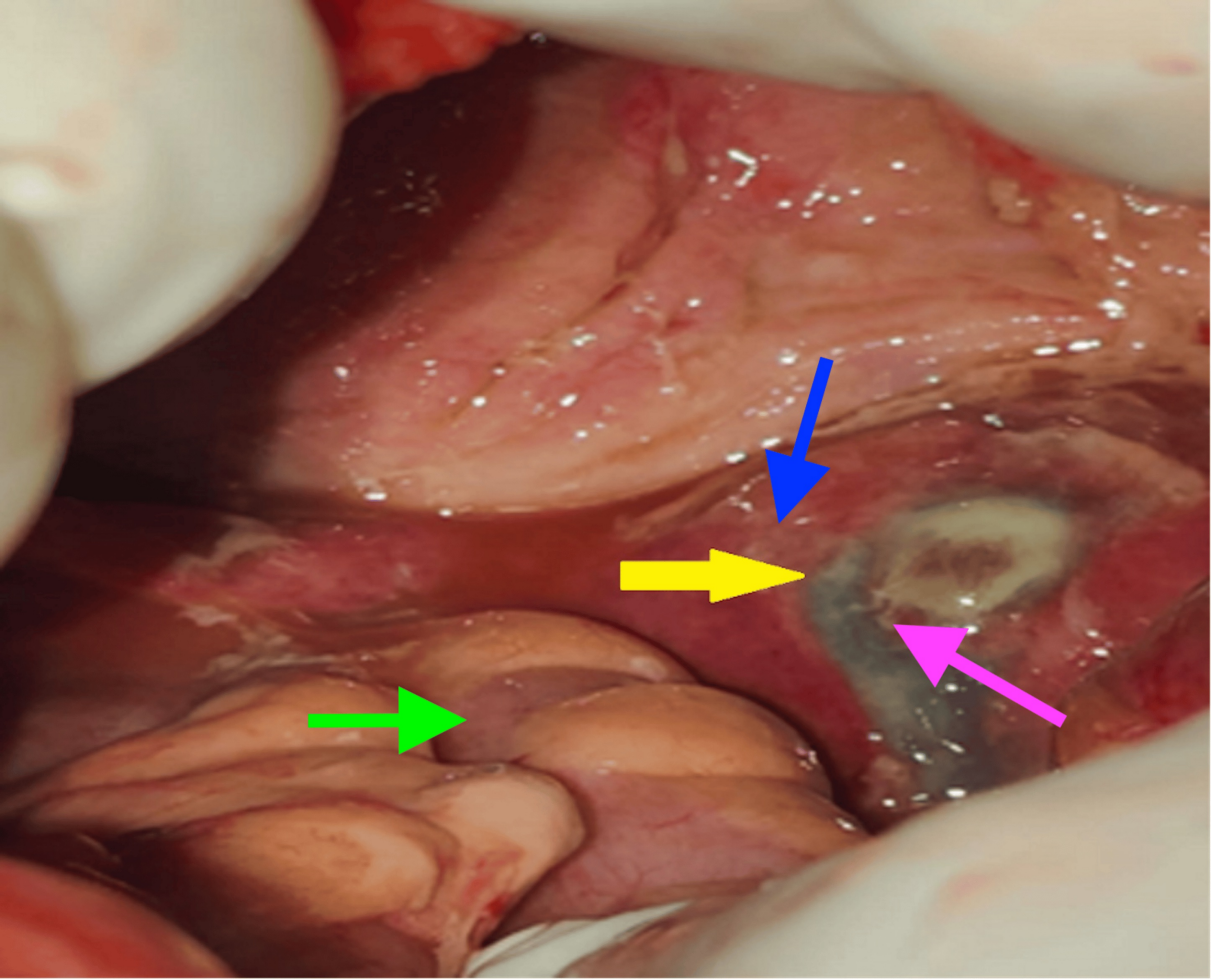
Uterus perforation on the anterior wall The yellow arrow shows perforated area with pus, green arrow shows intestine, blue arrow shows uterus, and pink arrow shows site of uterine perforation.

The patient was counselled for exploratory laparotomy, and written informed consent was obtained before commencement of the procedure. The intra-operative findings reported that there was no evidence of perforation in the bowel; however, peritonitis was present, and perforation of the fundus of the uterus 2x2 cm was observed. Additionally, uterus perforation was noted on the anterior wall with draining of pus (Figure 2).

**Figure 3:**
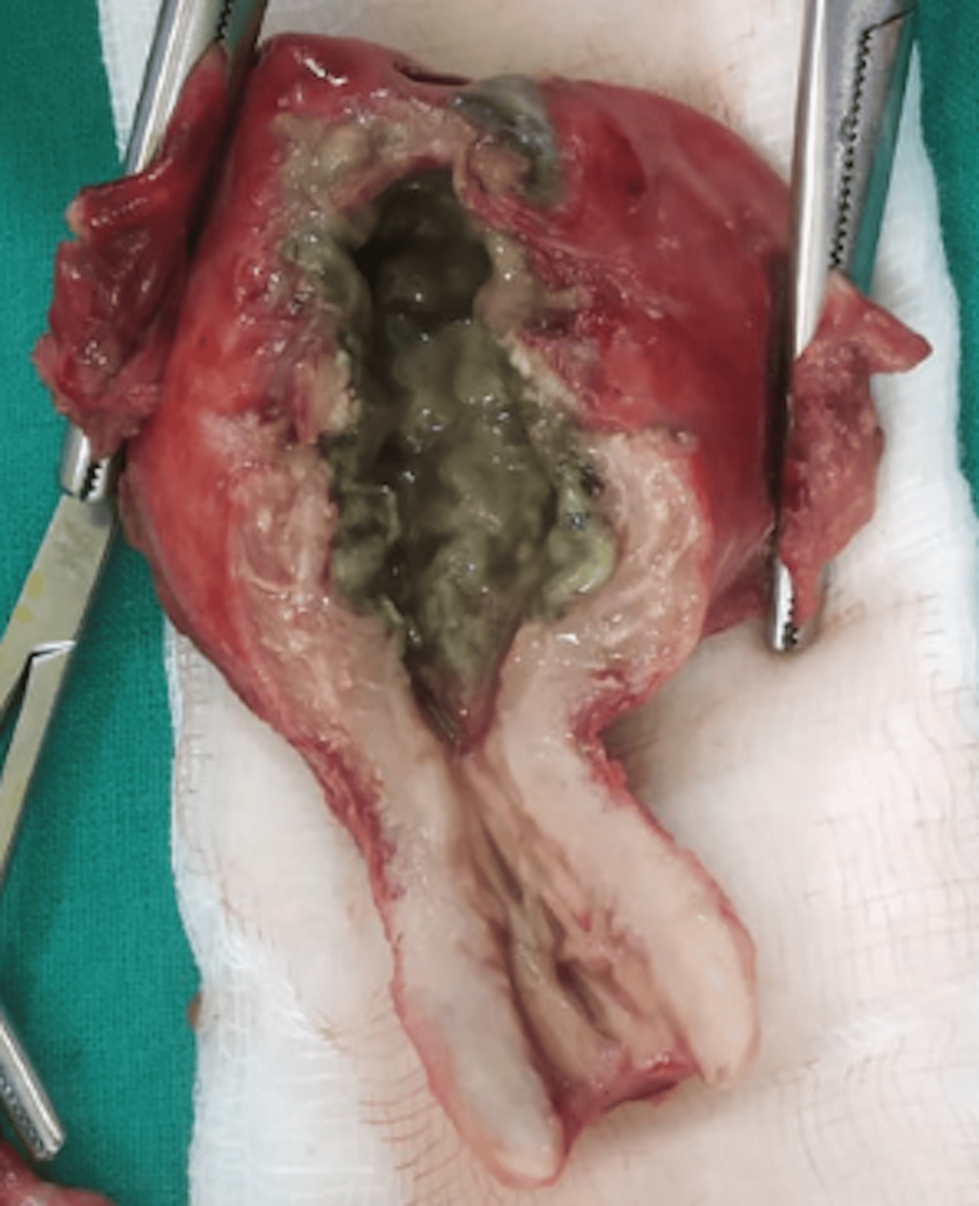
Hysterectomy specimen and perforated area

A total abdominal hysterectomy (TAH) with bilateral adnexectomy was performed and was directed for histopathological examination (HPE). A greenish area extending from the fundus to the junction of the cervix was observed, with an irregular brown area measuring 0.6 x 0.2 cm at the fundus, which is a site of perforation with a small polyp-like growth measuring 1 x 0.5 cm. The sectioned surface of both ovaries showed whitish areas (Figure 3).

**Figure 4:**
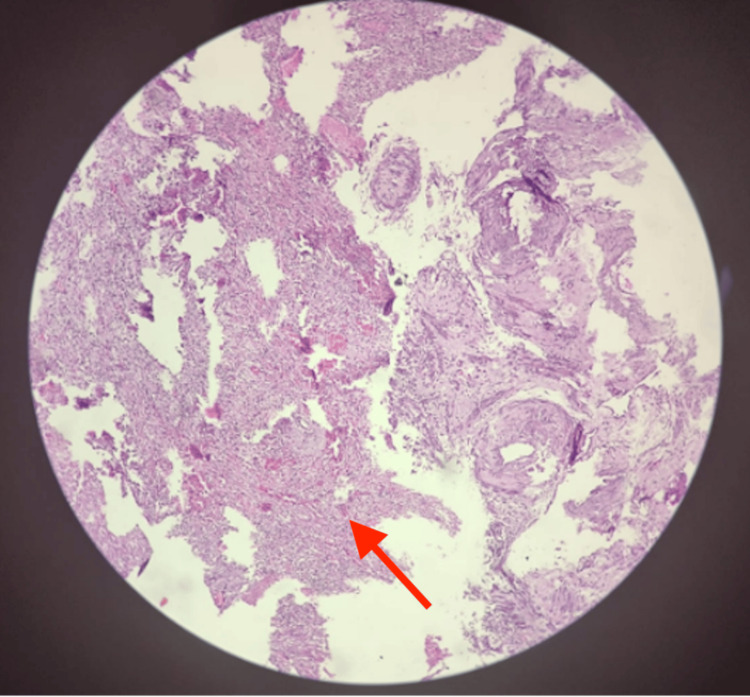
Histopathological examination slide, with the arrow pointing at thick bands of inflammation

The HPE of endometrial sections revealed thick bands of inflammation and necrosis replacing complete endometria. Moreover, endometrial glands and stroma were not observed (Figure 4).

Acute necrotizing inflammation of the uterus extending into the surrounding myometrium and up to the ectocervix below was observed, confirming the impression of uterine perforation and no evidence of malignancy or cellular atypia. Additionally, bilateral tubes and ovary were histologically normal.

Post-operatively, the patient developed tachypnoea and respiratory distress with respiratory hypoxemia on arterial blood gas, for which the patient was assisted with BiPAP for respiratory support and nebulization with duolin, budecort, and antibiotics. Chest X-ray was suggestive of mild pleural effusion of infective etiology. Moreover, the USG of the chest revealed bilateral moderate pleural effusion with underlying sub-segmental collapse and consolidatory changes. A USG-guided pleural tap was performed, and pleural fluid was sent for analysis, which revealed pale yellow, clear fluid, with 2-3 red blood cells/high power field, 30% polymorphs, and 70% lymphocytes, and no bacterial growth on culture was observed. Additionally, the cartridge-based nucleic acid amplification test (CB NAAT) for pleural fluid was negative. High-resolution CT impression reported pleural parenchymal bands in bilateral lung parenchyma, with moderate free fluid in the pleural cavity and underlying sub-segmental collapse.

The blood culture was found to be normal. The gram stain of pleural fluid results indicated the presence of pus cells and gram-negative rods. However, the pus culture of the pleural fluid reported no bacterial growth. The patient was managed in the surgical intensive care unit, with transfer to a gynecological unit for stabilization. The condition of the patient improved postoperatively; however, discharge from the suture site was noted. The wound swab culture reported *Staphylococcus aureus* sensitive to methicillin. Hence, as there were no other complications present, the patient recovered and was discharged with antibiotics.

## Discussion

Although postmenopausal women are more likely to experience uterine perforation, spontaneous uterine rupture in the context of pyometra is still extremely uncommon [7]. The typical age of a ruptured pyometra is 73.8 years, and the mortality rate following rupture ranges from 25% to 40% [8]. Since the uterus is an intraperitoneal structure, a surgical emergency may result from its rupture owing to an underlying infection and pus leakage into the abdominal cavity. According to a prior study, 77% of patients had uterine perforations at the fundus [9]. Similarly, in the present case, perforation of the fundus of the uterus 2x2 cm was observed, and, additionally, uterus perforation was noted on the anterior wall with draining of pus.

Pyometra resulting from malignancy demonstrates a poor prognosis and therefore should be the first differential diagnosis [10]. Similarly, in the present case, the patient was postmenopausal; however, the patient did not exhibit the presence of IUD, had no history of dilatation curettage operations and endometrial biopsy before, and had no intraoperative evidence of malignancy. Thus, cervix stenosis and postmenopausal changes, which cause discharge stagnation and anaerobic infection that results in fundus rupture, are the most probable causes of pyometra [6].

The comorbidities that have been related to an increased risk of rupture involve obesity, immobility, poor hygiene, type 2 diabetes mellitus, malnutrition, increased sexual activity, underlying fecal or urinary incontinence, cervical insufficiency, immunocompromised, and genital atrophy. However, the present case did not report any of these comorbidities. The flora of the genitourinary tract, including *Escherichia coli*, *Bacteroides*, *Streptococcus*, and other anaerobes, are the most frequently occurring organisms in pyometra [6]. However, in the present case, pus cultures showed the presence of Gram-negative rods, and the pus culture reported no bacterial growth.

Generalized abdominal pain, postmenopausal bleeding, and purulent vaginal discharge, accompanied by fevers, diarrhea, vomiting, and generalized weakness, are typical presentations of pyometra [11]. Additionally, an X-ray of the chest demonstrates pneumoperitoneum if there is uterine rupture [11]. These presentations corresponded with the symptoms reported by the patient in the present case consisting of pain in the abdomen (periumbilical) and fever for four days associated with nausea and vomiting. However, in the present case, the chest X-ray was found to be normal, and pneumoperitoneum was confirmed by CT of the abdomen with multiple air pockets, collapsed ileal loops, and moderate free fluid in the pelvis and abdomen, strongly indicating an intestinal perforation.

The primary treatment for spontaneous uterine rupture due to pyometra involves performing a total hysterectomy with bilateral salpingo-oophorectomy, administering broad-spectrum antibiotics, conducting extensive abdominal lavage, and ensuring postoperative ICU care for close monitoring [6]. Similarly, in the present case, an exploratory laparotomy was performed, which confirmed uterine perforation, for which TAH and bilateral removal of fallopian tubes and ovaries were performed. Hence, when the perforation is not linked with the malignancy, the prognosis is better [12].

## Conclusions

Uterine perforation caused by pyometra, leading to peritonitis and acute abdomen in postmenopausal women, is rare but associated with a high risk of morbidity and mortality. A thorough and detailed patient history is essential for an accurate diagnosis of the condition. Therefore, early diagnosis and appropriate and adequate management are crucial to avoid complications due to high chances of morbidity and mortality specifically in postmenopausal and comorbid patients. Additionally, spontaneous abdominal rupture of the uterus should be included as a differential diagnosis in elderly postmenopausal women presenting with acute abdominal pain, as it can be life-threatening. Furthermore, TAH with bilateral salpingo-oophorectomy, broad-spectrum antibiotic therapy, and comprehensive peritoneal lavage along with exploratory laparotomy or laparoscopy should be considered as the choice of treatment.
